# Quantitative assessment of heart-brain coupling in neonates with hypoxic-ischemic encephalopathy

**DOI:** 10.3389/fnetp.2026.1889331

**Published:** 2026-09-07

**Authors:** Danylo Reznichenko, Alina Frunza, Anastasiya Babintseva, Yevhenii Karpliuk, Oleksandr Romanchuk, Anton Popov, Kateryna Ivanko, Illya Chaikovsky

**Affiliations:** 1 Department of Electronic Engineering, Igor Sikorsky Kyiv Polytechnic Institute, Kyiv, Ukraine; 2 Scientific Department with an Innovative Development Sector, Bukovinian State Medical University, Central Municipal Clinical Hospital, Chernivtsi, Ukraine; 3 Department of Pediatrics, Neonatology and Perinatal Medicine, Bukovinian State Medical University, Chernivtsi, Ukraine; 4 Department of Internal and Family Medicine, Lesya Ukrainka Volyn National University, Lutsk, Ukraine; 5 Faculty of Applied Sciences, Ukrainian Catholic University, Lviv, Ukraine; 6 V. M. Glushkov Institute of Cybernetics of the NAS of Ukraine, Kyiv, Ukraine

**Keywords:** ECG, EEG, HIE, HRV, neonatal monitoring, network physiology, neuro-cardiac coupling, sarnat staging

## Abstract

Hypoxic-ischemic encephalopathy (HIE) remains a critical challenge in neonatal intensive care, requiring precise bedside monitoring to mitigate long-term neurological morbidity. While individual assessment of cerebral and cardiovascular activity is standard, the dynamic interaction between these systems provides untapped diagnostic value. This pilot study investigates neuro-cardiac coupling using synchronized long-term EEG and ECG recordings, focusing primarily on a core subset of 61 neonates classified by clinical HIE severity (Sarnat Stage 1 vs. Stage 2). We employed cross-modal analysis to evaluate the relationship between the amplitude-integrated EEG (aEEG) envelope and heart rate variability (HRV) metrics. Our results demonstrate that neuro-cardiac interaction is significantly modulated by HIE severity and electrocortical background patterns. Neonates with healthy continuous normal voltage (CNV) patterns exhibited robust neuro-cardiac synchronization (median Spearman’s r=0.22 ), which was significantly reduced by approximately 60% in patients with abnormal EEG backgrounds (median Spearman’s r=0.09 , p=0.015 ). To ensure robust translation, machine learning models were evaluated across diverse parameter grids. While isolated hyperparameter configurations yielded peak performance metrics–such as an overall maximum accuracy of 90.22% using combined cardiocycle and spectral ECG features with XGBoost–the feature space demonstrated strong, un-biased baseline stability, yielding reliable average accuracies across window lengths (e.g., 77.27% mean $F_{1}$ -score for EEG-based Logistic Regression and 72.73% mean $F_{1}$ for ECG-based XGBoost). These findings suggest that multi-modal coupling metrics offer enhanced physiological insight for automated HIE grading in a pilot setting.

## 1 Introduction

Hypoxic-ischemic encephalopathy (HIE) is a significant cause of neonatal mortality and long-term neurological morbidity, resulting from systemic oxygen deprivation and reduced cerebral blood flow during the perinatal period. To assist in clinical management and prognosis, the Sarnat staging system–introduced by Sarnat and Sarnat in 1976 – remains the gold standard for classifying the severity of encephalopathy into three distinct clinical stages (Sarnat and Sarnat, 1976). Stage 1 (mild) is typically characterized by hyper-alertness, irritability, and sympathetic activation; Stage 2 (moderate) involves lethargy, suppressed primitive reflexes, and the onset of seizures; while Stage 3 (severe) presents as profound coma, absent reflexes, and respiratory failure. Early and accurate differentiation between these stages, particularly the transition from mild to moderate injury, is clinically vital as it dictates the window for neuroprotective interventions such as therapeutic hypothermia.

The differentiation between Sarnat Stage I (mild HIE) and Sarnat Stage II (moderate HIE) remains one of the greatest challenges in neonatal neurology because the clinical manifestations of these stages frequently overlap. Neurological signs are often subtle and subjective, while therapeutic interventions such as hypothermia or sedation may further complicate early assessment. Since eligibility for therapeutic hypothermia depends on timely identification of moderate HIE, accurate discrimination between these stages is of critical clinical importance.

Although amplitude-integrated EEG (aEEG) is the standard bedside tool for monitoring cerebral function, the differences between mild and moderate HIE are often too subtle for reliable visual interpretation alone. Likewise, heart rate variability (HRV) derived from ECG provides complementary information on autonomic nervous system dysfunction but cannot fully characterize brain injury severity when used independently. Machine learning offers the opportunity to integrate quantitative features extracted from both aEEG and ECG, identifying complex multimodal patterns that are difficult to recognize using conventional analysis. Such approaches may improve the objective differentiation between Sarnat Stages I and II, enabling earlier diagnosis and supporting timely therapeutic decision-making.

Multiple studies have demonstrated measurable coupling between neonatal brain activity (EEG) and cardiac signals (ECG/HRV) in hypoxic-ischemic and seizure contexts. For example, slow-wave bursts in preterm EEG are synchronized with transient accelerations of heart rate (Pfurtscheller et al., 2005). Neonatal seizures (often due to HIE) frequently induce autonomic changes–Doyle et al. found that instantaneous heart rate (HR) changes were significantly correlated with EEG amplitude during seizures (Doyle et al., 2008). Likewise, measures of HR variability (HRV) correlate strongly with EEG background grade in HIE: Goulding et al. report that time-domain indices such as SDNN (standard deviation of the NN intervals) and TINN (triangular interpolation of the NN interval histogram), as well as frequency-domain components—low-frequency (LF) and high-frequency (HF) power—all decrease as EEG abnormalities worsen ( |r|≈0.48 –0.67, p<0.05 ) (Goulding et al., 2015), and Pavel et al. show that combining HRV features (time, frequency, entropy) with clinical data predicts EEG severity, achieving an Area Under the Receiver Operating Characteristic curve (AUC) ≈0.84 (Pavel et al., 2023). Other work has found EEG–ECG coupling in sleep states (e.g., increased EEG δ -power in quiet sleep coincides with lower HR variability) (Govindan et al., 2026) and in quantitative HRV entropy differences between seizure and non-seizure newborns (Frassineti et al., 2021). In sum, EEG band-powers ( δ , θ , α , β ) and EEG-derived metrics (RMS, burst rate) can correlate with ECG-derived features (meanNN, the mean of the NN intervals; SDNN; RMSSD, the root mean square of successive differences; LF, HF, LF/HF, entropy), suggesting that combined EEG + ECG analysis may improve HIE assessment.

From the broader perspective of Network Physiology, the human organism is conceptualized as an integrated network of interacting organ systems, where complex macro-states emerge from continuous, dynamical cross-talk between individual subsystems. Within this paradigm, the central nervous and cardiovascular systems form a critical, highly coordinated brain-heart network. Investigating the multi-scale coupling between electrocortical rhythms (EEG) and autonomic cardiac regulation (ECG/HRV) in neonates is therefore essential. In the context of hypoxic-ischemic injury, this approach allows us to observe how systemic stressors disrupt global network topology, shifting the system from flexible, homeostatic interactions to pathological states. Consequently, assessing this brain-heart axis offers a holistic window into the infant’s physiological network integrity that cannot be achieved through isolated, unimodal assessments (Ivanov, 2021; Antonacci et al., 2023).

The aim of the study is to investigate the characteristics of bioelectrical brain activity and heart-brain coupling in newborns with HIE Stage I and II according to the Sarnat-Sarnat scale. We review key findings and then outline recommended experimental analyses: preprocessing, feature extraction (EEG power spectra and HR/HRV metrics), correlation and statistical testing, visualization, and predictive modeling (e.g., logistic regression, random forests with AUC/sensitivity).

### 1.1 Neonatal seizures and HR changes

Neonatal seizures, especially those due to HIE, often trigger autonomic responses. Greene et al. combined EEG and ECG for seizure detection and note that “neonatal seizures are often associated with changes in heart and respiration rate” (Greene et al., 2007). In a cohort of 10 term infants (633 labeled seizures), adding ECG to multichannel EEG improved seizure detection rates (e.g., 98% of seizures detected vs. ∼ 81% with EEG alone) (Greene et al., 2007). Doyle et al. (EMBC 2008) quantitatively demonstrated this coupling: in 12 neonates, changes in instantaneous HR were significantly correlated (p<0.05) with the root-mean-square (RMS) EEG amplitude during seizures – 100% of patients showed this effect (in 83% of seizures) (Doyle et al., 2008). These studies imply that transient EEG ictal events coincide with heart rate accelerations or decelerations. Pfurtscheller et al. found the same phenomenon in preterms: “EEG bursts of slow waves during trace alternant are coupled with an acceleration of the HR” (Pfurtscheller et al., 2005). Thus, specific EEG features such as burst onsets or RMS increases tend to co-occur with ECG/HR changes, providing concrete correlated feature pairs (e.g., slow-wave burst ↔ HR acceleration, EEG RMS ↔ RR-interval drop).

### 1.2 EEG vs. HRV in HIE

Beyond seizures, studies have examined baseline EEG background against HR variability in HIE infants. Goulding et al. (2015) showed that standard HRV metrics correlate with EEG grade of encephalopathy. In 46 term infants with HIE (graded normal/mild/moderate/severe by EEG), SDNN (std. dev. of RR) correlated negatively with EEG severity (r≈−0.62) , as did TINN (r≈−0.65) , LF power (r≈−0.67) and HF power (r≈−0.60) (Goulding et al., 2015). In other words, worse EEG (flatter background) was linked to lower HRV. Pavel et al. (Front. Pediatr. 2023) systematically used HRV to predict early EEG grade in HIE. In 120 infants, logistic models using HRV (time, freq, entropy) achieved AUC ≈0.84 for normal/mild vs. moderate/severe EEG (improving to ≈0.90 with clinical covariates) (Pavel et al., 2023). This underscores that HRV features (meanNN, SDNN, LF, HF, etc.) quantitatively reflect EEG-based HIE severity.

### 1.3 Developmental and sleep-state coupling

Even in healthy neonates, EEG–ECG coupling appears. Govindan et al. (2026) found that in 68 preterm infants, active vs. quiet sleep states exhibited distinct EEG and HR patterns: delta-band EEG power was significantly higher in quiet sleep (QS) than active sleep (AS) (p<0.05) , while HR variability (range) was lower in QS (Govindan et al., 2026). An ROC analysis on HR range separated AS vs. QS (AUC ≈0.88 ). In sum, state-dependent EEG rhythms ( ↑δ in QS) correspond to autonomic shifts (reduced HR range) (Govindan et al., 2026). These findings imply EEG–ECG feature correlations linked to maturation of brain–heart control.

### 1.4 Summary of correlated features

Table 1 provides a structured overview of representative studies investigating the relationship between EEG and ECG characteristics in neonates. Within the Network Physiology framework, these cross-modal variations are increasingly quantified via advanced time-series coupling frameworks that capture how different physiological rhythms co-vary across micro- and macro-scales (Garcia-Retortillo et al., 2026).

**TABLE 1:** Key studies of neonatal EEG–ECG feature correlation.

Study (Year)	Population and signals	N	Correlated features	Main result (effect size)	DOI
Greene et al. (2007)	10 term neonates; EEG (7–12ch) + ECG (NICU data)	10	Seizure epochs (EEG) vs. HR changes	Combined EEG + ECG detector GDR ∼ 98% (vs. 81% EEG-only) (Greene et al., 2007)	https://doi.org/10.1016/j.clinph.2007.03.022
Doyle et al. (2008)	12 neonates; EEG + ECG (seizure monitoring)	12	EEG RMS vs. iHR change	100% patients had EEG–HR correlation during seizures (Doyle et al., 2008)	https://doi.org/10.1109/IEMBS.2008.4650333
Pfurtscheller et al. (2005)	Preterm infants (mean CA = 36w); EEG (tracé alternant) + HR	∼ 9	EEG slow-wave bursts vs. HR accel	Slow-wave bursts tightly coincide with transient HR accelerations (Pfurtscheller et al., 2005)	https://doi.org/10.1016/j.braindev.2005.01.007
Goulding et al. (2015)	Term HIE infants; EEG grade + ECG	46	HRV (SDNN, LF, HF, etc.) vs. EEG grade	HRV metrics negatively correlated with EEG severity ( r≈−0.48 to −0.67 ) (Goulding et al., 2015)	https://doi.org/10.1038/pr.2015.28
Pavel et al. (2023)	Term HIE infants; EEG grade + ECG	120	HRV (time, freq, entropy)	HRV model AUC = 0.84 for predicting EEG grade (Pavel et al., 2023)	https://doi.org/10.3389/fped.2022.1016211
Frassineti et al. (2021)	Neonatal seizures; ECG (Helsinki NICU dataset)	52	HRV multiscale entropy	Seizure vs. non-seizure groups differ in HRV entropy (p<0.05) (Frassineti et al., 2021)	https://doi.org/10.3390/bioengineering8090122
Govindan et al. (2026)	Preterm sleep study; EEG + ECG	68	Sleep state (EEG δ -power vs. HR range)	QS: ↑δ -power vs. AS, distinct HR range (AUC ≈0.88 ) (Govindan et al., 2026)	https://doi.org/10.1038/s41598-025-31873-7

Abbreviations: SDNN: standard deviation of NN, intervals; LF/HF: low-frequency/high-frequency; iHR: instantaneous heart rate; CA: conceptional age; GDR: gross detection rate; RMS: root-mean-square.

The first approach, as characterized by foundational work in acute monitoring, focuses on the immediate temporal synchronization between localized cerebral events and autonomic cardiac responses. This includes the close alignment of heart rate fluctuations with electrographic ictal configurations (Greene et al., 2007), the significant correlation between instantaneous heart rate changes and transient EEG burst amplitudes (Doyle et al., 2008), and the consistent pairing of slow-wave trace alternant bursts with autonomic accelerations (Pfurtscheller et al., 2005). Conversely, the second approach shifts focus toward steady-state macro-dynamics, demonstrating how continuous HRV indices systematically covary with global electrocortical background degradation (Goulding et al., 2015), sleep-state organization (Govindan et al., 2026), and macro-scale encephalopathy severity classifications (Pavel et al., 2023). Furthermore, the application of non-linear frameworks, such as multiscale entropy, highlights that these cross-modal variations reflect deeper, structural alterations within the infant’s underlying neuro-cardiac networks (Frassineti et al., 2021).

Collectively, these studies provide consistent evidence of a close interaction between cerebral electrical activity and autonomic cardiac regulation in newborns. The documented associations—spanning both micro-scale temporal adjustments and macro-scale feature correlations—establish a strong physiological basis for multimodal EEG–ECG analysis. Crucially, they serve as the theoretical reference for defining the experimental objectives of the present study, validating our feature space composition and providing the framework necessary to investigate neuro-cardiac coupling patterns across Sarnat Stage 1 and Stage 2 encephalopathy.

## 2 Materials and methods

### 2.1 Data description

The dataset utilized in this study was obtained from the Neonatal Intensive Care Unit of the Central Municipal Clinical Hospital, which is a clinical site of the Bukovinian State Medical University (Chernivtsi, Ukraine). Data collection was performed in strict accordance with the Declaration of Helsinki (1964–2013), ICH GCP (1996), Decree of the Ministry of Health of Ukraine № 690 from 23.09.2009, and the Protocol of scientific research of the Commission of Biomedical Ethics of the Bukovinian State Medical University. Informed written consent was obtained from the parents, setting out the purpose, objectives, and methods of the study.

**FIGURE 1:**
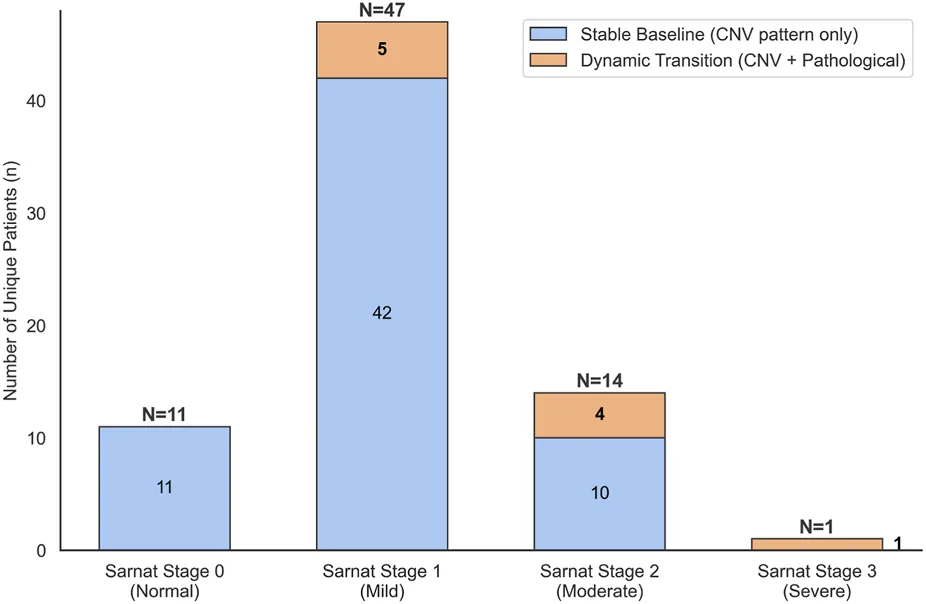
Distribution of unique neonates across clinical Sarnat severity stages paired with intra-patient electrocortical background pattern characteristics. The stacked orange segments visually isolate the specific nested subset of infants who demonstrated dynamic state transitions - contributing both normal continuous baseline activity (CNV) and acute compromised recordings (DNV, BS, CLV, FT) over the course of long-term monitoring.

Table 2 shows the total number of children and EEG recordings included in the overall study database. Additionally, the explicit stratification of unique patients within the ECG-matched multi-modal analytical cohort across clinical Sarnat stages and their corresponding longitudinal electrocortical background pattern trajectories is illustrated in Figure 1. Beyond signal-derived metrics, the dataset includes clinical features such as gestational age (GA) and sex.

**TABLE 2:** Dataset distribution across Sarnat stages, highlighting the subset of records containing synchronous ECG data.

Sarnat stage	Total EEG records (patients)	Subgroup with ECG (patients)
Stage 0	15 (14)	11 (11)
Stage 1	156 (118)	64 (47)
Stage 2	122 (62)	28 (14)
Stage 3	27 (7)	5 (1)

In accordance with the objective, the first stage of the statistical analysis included 180 newborns which were stratified into two groups: 118 infants with Stage 1 and 62 infants with Stage 2 of HIE according to the Sarnat-Sarnat classification. In this patient cohort, an autonomous quantitative analysis was performed on 289 available EEG (qEEG) recordings with a sampling frequency of 500 Hz. Of these, 160 EEG recordings were obtained from newborns with Stage 1 HIE, and 129 recordings were obtained from newborns with Stage 2 HIE. The gestational age of the newborns ranged from 26 to 43 weeks. Children of the male sex predominated in both subgroups: 55.9% in Stage 1% and 64.5% in Stage 2.

In the second stage of the study the primary multi-modal cross-coupling and clinical HIE staging framework focuses specifically on the sub-cohort of neonates with fully synchronized, simultaneous EEG and ECG telemetry ( n=61 unique patients; 47 Stage 1, 14 Stage 2), which was captured at a synchronized sampling frequency of 200 Hz. In this subgroup 64 EEG + ECG recordings were obtained from newborns with Stage 1 HIE, and 28 recordings were obtained from newborns with Stage 2 HIE.

Notably, while the full database covers a wide GA spectrum, this core ECG-matched analytical cohort (n=61) naturally restricted itself to term and near-term neonates with a GA range of 34–41 weeks. This structural composition inherently isolates the analysis from gestational maturity confounders, as neonates within this specific developmental window exhibit fully established baseline electrocortical patterns and consistent, mature sleep-wake cycling characteristics (Hellström-Westas et al., 2006).

In the multimodal subgroup, boys also predominated across the various stages of HIE: 55.3% at Stage 1% and 85.7% at Stage 2. It should be noted that this is an exploratory pilot study with a constrained sample size in the minority class, stratified matching by sex was not feasible, and sex was treated as an unstratified clinical covariate.

In addition, background patterns in the multimodal subgroup were tracked using 108 distinct EEG and ECG recording segments derived from 73 unique patients. All 73 infants contributed normal baseline recordings (CNV), while a subset of 10 infants exhibited dynamic state transitions, additionally contributing all recordings classified as pathological (DNV, BS, CLV, FT).

Data annotation for hypoxic-ischemic encephalopathy (HIE) severity was performed using clinical Sarnat-Sarnat staging (Sarnat and Sarnat, 1976). The aEEG assessment was conducted in accordance with the classification by Hellström-Westas L., distinguishing the following patterns: Continuous Normal Voltage (CNV), Discontinuous Normal Voltage (DNV), Burst-Suppression (BS), and Continuous Low Voltage (CLV) (Hellström-Westas et al., 2006). Due to its singular occurrence, the Flat Trace (FT) pattern was consolidated into the CLV category.

All physiological signals were recorded in standard BioSemi Data Format (BDF) using a clinical-grade “Simplex EEG-CMF” amplitude-integrated electroencephalograph (UKRMEDSPEKTR LLC, Kharkiv, Ukraine) bedside acquisition monitor. The EEG data includes two channels (F3-P3, F4-P4), while the ECG was recorded via channel C3-C4 (equivalent to V3-V4). Beyond signal-derived metrics, the dataset includes clinical features such as gestational age and sex, which were incorporated into the classification models. Recording durations were highly variable, reflecting diverse clinical monitoring requirements in the neonatal intensive care unit (NICU), with signal lengths ranging from a minimum of 1,120 s ( ≈ 19 min) to a maximum of 46,445 s ( ≈ 13 h), with a mean recording length of 73 min.

The signals were processed in their continuous form rather than being artificially truncated into arbitrary short-term epochs, preserving macro-scale dynamics and long-term trends like sleep-wake transitions. To systematically mitigate potential analytical biases introduced by this signal length variation, mathematical normalization was implemented at two levels. First, all continuous statistical coupling metrics, cross-correlations, and temporal lag shifts were calculated strictly within standardized, localized 5-min sliding windows and subsequently aggregated via patient-level averaging. Second, for the machine learning classifiers, long-term features were transformed into robust summary statistics and time-invariant averages (such as aggregate cardiocycle morphological descriptors), ensuring that the final feature representations are mathematically normalized and invariant to the total recording duration. Due to significant class imbalances–specifically, 85% of signals containing ECG data exhibited the CNV pattern–the classification and feature significance analysis focused exclusively on Sarnat levels 1 and 2. This decision was further supported by clinical expert consensus that healthy neonates (level 0) and those with severe HIE (level 3) are readily identifiable through standard clinical assessment, and these categories were represented by an insufficient number of signals for robust statistical modeling in this specific cohort. The cohort distribution across all levels is detailed in Table 2.

Rather than applying aggressive, threshold-based manual artifact rejection—which risks introducing selection bias by preferentially removing segments containing high motor activity or clinical seizures—signal quality across all modalities was managed architecturally through pipeline robustness. This approach assumes that ambient noise and transient artifacts are distributed non-differentially across clinical groups and should not systematically distort class-specific comparisons.

To handle real-world NICU interference across different signal types, distinct strategies were deployed. For the EEG stream, resilience against localized noise and transient emissions was achieved through targeted bandpass filtering combined with median-based window aggregations and time-invariant statistical descriptors during feature extraction. For the ECG stream, the determination of heart rate variability parameters specifically relied on the built-in ectopic beat detection and outlier correction protocols provided by the pyHRV library. Together, these methods ensure that the final computed multimodal metrics reflect true macroscopic physiological dynamics rather than localized recording artifacts.

### 2.2 Signal preprocessing

#### 2.2.1 qEEG/aEEG preprocessing

To compensate for minor inter-channel hardware sampling jitter and localized phase propagation delays—which introduced micro-alignments of up to several data points in the raw recordings—EEG channels were synchronized by temporally shifting signals to maximize the absolute value of their cross-correlation. Asymmetry and average signals were subsequently derived to ensure artifact robustness. Signals were resampled to 100 Hz using FFT-based methods and processed with a 50 Hz notch filter. Artifact rejection was handled via zero-imputation for values exceeding 500μV .

The amplitude-integrated EEG (aEEG) was generated based on the foundational algorithm described by Zhang and Ding (Zhang and Ding, 2013). While proprietary median-based smoothing adjustments were incorporated to enhance artifact resistance, the core pipeline—specifically the initial amplitude clipping, asymmetric bandpass filtering (0.5–15 Hz), amplitude envelope extraction via the Hilbert transform, and subsequent semi-logarithmic amplitude compression—strictly adheres to the established international consensus and mathematical standards for digital aEEG derivation (Chen et al., 2021; Hellström-Westas et al., 2006). Smoothing was performed using rolling windows of varying sizes (15, 30, 60, 120, 180, and 300 s). These specific temporal lengths were selected to capture a full spectrum of physiological dynamics, ranging from rapid autonomic micro-fluctuations (15–30 s) to stable, macro-scale metabolic and sleep-state drifts (180–300 s). From these windows, the $10^{th}$ (lower), $90^{th}$ (upper), and $50^{th}$ (median) quantiles were extracted. The inclusion of the median trace represents a deviation from standard aEEG methodology, providing a robust central tendency metric that is less sensitive to transient artifacts than the standard mean. Finally, the signals were exponentiated to return the traces to the original microvolt (μV) scale.

#### 2.2.2 ECG and HRV preprocessing

ECG signals were preprocessed using the NeuroKit2 ecg_clean method to ensure high signal-to-noise ratios for both morphological and heart rate analysis (Makowski et al., 2021). The cleaning pipeline consisted of two primary stages: first, a $5^{th}$ -order Butterworth high-pass filter with a 0.5 Hz cutoff frequency was applied to eliminate baseline wander; second, a notch filter at 50 Hz was utilized to suppress powerline interference. Following the initial signal preprocessing, the BioSPPy library (Carreiras et al., 2015) was employed strictly for the task of R-peak detection to isolate raw RR intervals.

**FIGURE 2:**
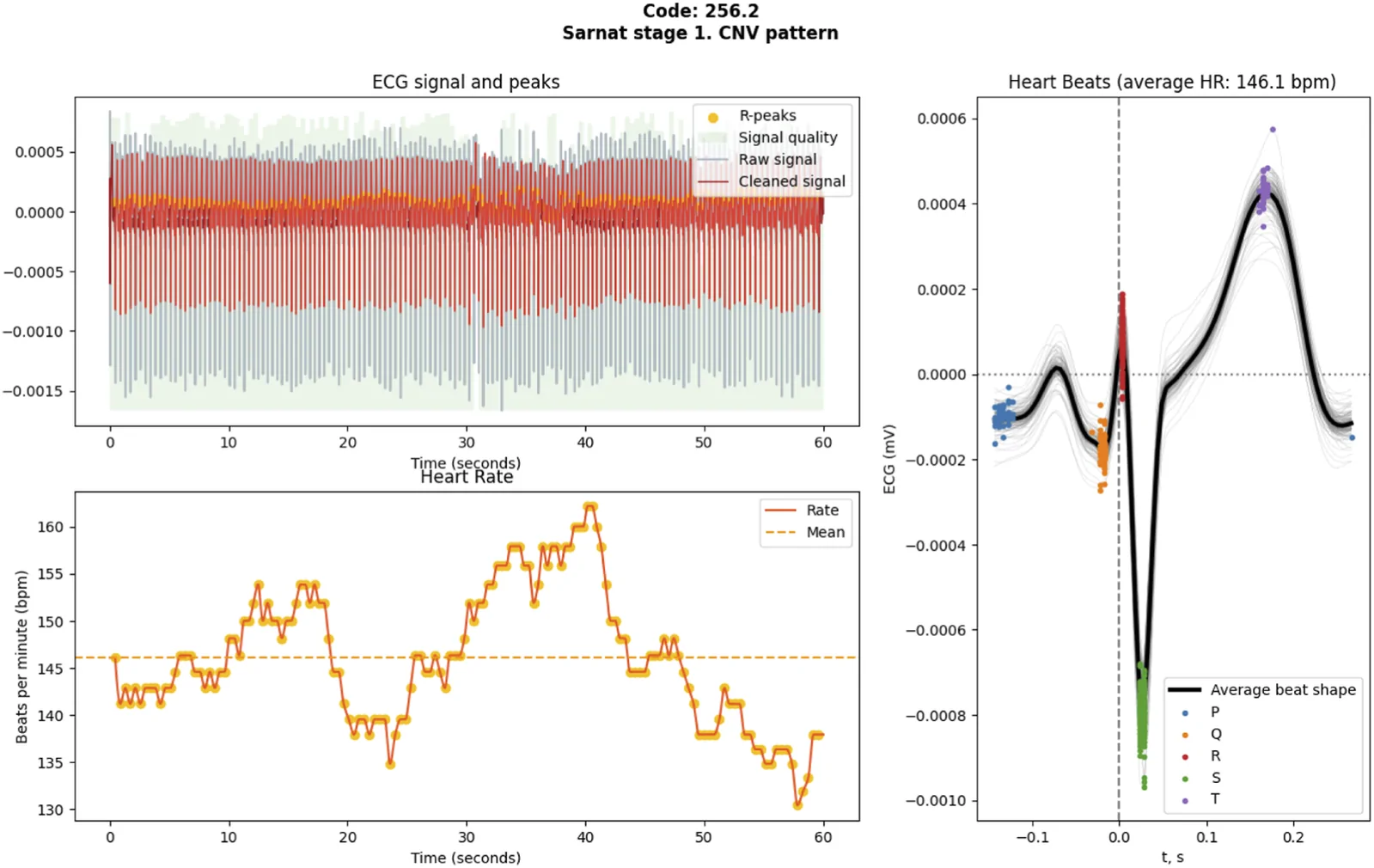
Examples of neurokit2 signal preprocessing for a Sarnat stage 1 patient: raw and cleaned signals, heart rate variability trace, and overlapped cardiocycles.

**FIGURE 3:**
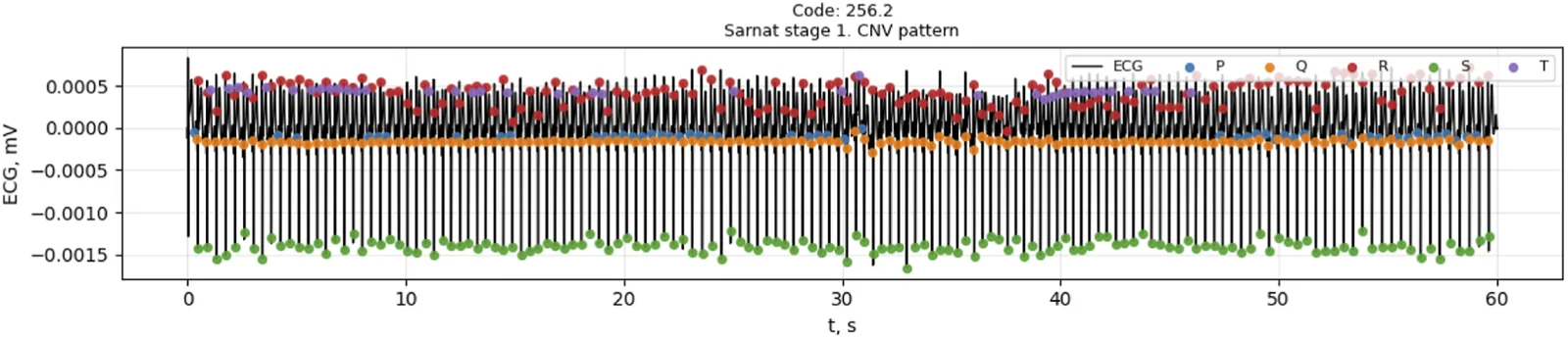
Marked ECG peaks including P, Q, R, S, and T waves.

For detailed morphological analysis, the preprocessed ECG signals were partitioned into individual cardiocycles using the ecg_segment method from NeuroKit2 (an open-source Python Toolbox for Neurophysiological Signal Processing) (Makowski et al., 2021). This process involved defining a fixed time–window around each R-peak to isolate the P-QRS-T complex. These segments were then synchronized and aggregated to derive average heartbeat templates and local morphological features–such as peak amplitudes and segment durations–for each patient recording. A representative visualization of this continuous preprocessing workflow, including the raw and cleaned streams alongside the resulting heartbeat templates, is illustrated in Figure 2. The precise localization and annotation of the individual P, Q, R, S, and T wave landmarks within these segments are explicitly highlighted in Figure 3.

### 2.3 Feature extraction

#### 2.3.1 qEEG and aEEG features

A total of 25 features were extracted for each aEEG channel and envelope, including standard statistical moments, spectral power ratios, and descriptors of signal complexity and discontinuity. Robust thresholds for trace classification were defined using the 10th $\left(Q_{0.10}\right)$ and 90th $\left(Q_{0.90}\right)$ quantiles to ensure artifact resistance (Equations 1–3):

$$T_{\mathrm{upper}}=0.9\cdot Q_{0.90}+0.1\cdot Q_{0.10}$$ (1)

$$T_{\mathrm{lower}}=0.1\cdot Q_{0.90}+0.9\cdot Q_{0.10}$$ (2)

$$T_{\mathrm{middle}}=\frac{Q_{0.90}+Q_{0.10}}{2}$$ (3)

To capture the complexity and morphological dynamics of the signal, we calculated the Hjorth parameters–Activity, Mobility, and Complexity–defined as defined in Equations 4–6:

$$\text{Activity}=\sigma_{x}^{2}$$ (4)

$$\text{Mobility}=\frac{\sigma_{x^{\prime }}}{\sigma_{x}}$$ (5)

$$\text{Complexity}=\frac{\text{Mobility}\left(x^{\prime }\right)}{\text{Mobility}\left(x\right)}=\frac{\sigma_{x^{\prime \prime }}/\sigma_{x^{\prime }}}{\sigma_{x^{\prime }}/\sigma_{x}}$$ (6)

where $\sigma_{x}^{2}$ denotes the variance of the signal x , and $x^{\prime }$ and $x^{\prime \prime }$ represent the first and second derivatives of the signal, respectively. Furthermore, non-linear dynamics were quantified using permutation entropy and spectral entropy to characterize the signal’s information content and irregularity. Finally, to assess signal discontinuity, the Burst Rate (BR) and Inter-Burst Interval (IBI) were computed; a “burst” was operationally defined as signal segments exceeding the 95th percentile amplitude threshold. The Burst Rate was quantified as the frequency of burst onsets per minute, while the IBI was determined as the average temporal duration (in seconds) between the onsets of consecutive bursts.

#### 2.3.2 Heart rate variability (HRV)

Heart rate variability (HRV) features were categorized into two distinct sets based on their extraction methodology: Standardized Feature Set derived via the pyHRV library (Gomes et al., 2019) and custom features computed directly from the cardiorythmogram (CRG). The pyHRV library was used to extract a standard suite of time-domain (e.g., SDNN, RMSSD, pNN50), frequency-domain (FFT-based power spectra), and non-linear metrics (Poincaré plot parameters, Sample Entropy). These features benefited from the library’s internal signal-checking and outlier-mitigation protocols, establishing a highly stable baseline for model training.

In parallel, a larger Extended Feature Set was programmatically derived from both the raw CRG and a version interpolated at 8 Hz using a cubic spline (CRG_interp). This set focused on high-resolution morphological and spectral characteristics, including.

- Statistical Descriptors: Higher-order moments (skewness, kurtosis) and range-based metrics (Max-Min).
- Wavelet Decomposition: A 3-level discrete wavelet decomposition was performed using a Daubechies 8 (db8) mother wavelet, with energy and statistical distributions computed across the resulting detail (cD1–cD3) and approximation (cA3) coefficients.
- Extended Spectral Bands: Relative and absolute energy ratios computed across five distinct bands: Ultra-Low-Frequency (ULF: <0.015 Hz), Very-Low-Frequency (VLF: 0.015−0.04 Hz), Low-Frequency (LF: 0.04−0.15 Hz), High-Frequency (HF: 0.15−0.40 Hz), and Very-High-Frequency (VHF: >0.40 Hz).

#### 2.3.3 ECG and cardiocycles

ECG signals were analyzed through a bank of filters targeting specific physiological rhythms, including T-wave (1–10 Hz), QRS complex (10–49 Hz), Baseline Wander (BW - below 0.5 Hz), Motion Artifact (2–22 Hz), and Electromyogram (EMG - above 50 Hz) noise. Integrating noise and artifact frequency ranges as features provides the model with critical context regarding signal quality, allowing it to weight clinical features more effectively based on the integrity of the recording. Furthermore, these ranges may capture indirect physiological indicators–such as increased muscle activity or motion artifacts–that can correlate with a neonate’s irritability or clinical distress level within the Sarnat scale. Cardiocycles were evaluated for morphological features such as PR/QT intervals and QRS symmetry, defined in Equation 7 as:

$$S_{\mathrm{QRS}}=\frac{R_{\mathrm{peak}}-P_{\mathrm{offset}}}{R_{\mathrm{offset}}-P_{\mathrm{offset}}}$$ (7)

Average heartbeat quality was determined using the NeuroKit2 distance-from-mean method. For a segmented heartbeat $C_{j}$ represented as a Z-standardized vector of length T , the quality Q is derived from the mean Z-score (Equation 8):

$$D_{j}=\frac{1}{T}\overset{T}{\underset{t=1}{\sum }}|Z_{j,t}|,\;Q=1-\text{Rescale}\left(|D_{j}|,\left[0,1\right]\right)$$ (8)

#### 2.3.4 Feature space composition and notations

To provide a transparent overview of the high-dimensional feature space, the structural composition of the extracted variables across all physiological modalities is summarized in Table 3. In total, a pool of 774 distinct features was evaluated. The EEG feature space (n=240) balances macro-scale background trends via amplitude-integrated EEG statistics alongside localized micro-fluctuations via quantitative spectral metrics. The cardiovascular feature space comprises heart rate variability (HRV) parameters (n=132) , basic statistical/spectral properties of the continuous ECG stream (n=130) , and fine-grained multi-scale cardiocycle morphological descriptors (n=272) tracking ventricular repolarization and atrial depolarization waveforms. Structurally, the HRV features encompass a standardized core suite (n=38) extracted via the open-source pyHRV framework (Gomes et al., 2019) to leverage its built-in outlier mitigation, alongside an extended feature set (n=94) capturing raw time-domain statistics, multi-band spectral densities, and discrete wavelet coefficients directly from raw and interpolated cardiorythmograms (CRG). Standard mathematical transformations (such as the arithmetic mean, standard deviation, skewness, and kurtosis) were utilized as descriptive summary aggregators across sliding windows; to maintain concise reporting, explicit textbook equations for these fundamental statistical operators are omitted.

**TABLE 3:** Summary of the extracted feature space across physiological domains.

Feature domain	Source signal/Channels	Primary extraction framework/References	Total features
EEG (aEEG)	Derived traces ( ${\text{EEG}}_{\mathrm{avg}}$ , ${\text{EEG}}_{\mathrm{asym}}$ )	Statistical descriptors of 4 envelope streams	200
EEG (qEEG)	Derived traces ( ${\text{EEG}}_{\mathrm{avg}}$ , ${\text{EEG}}_{\mathrm{asym}}$ )	Spectral power bands, Hjorth parameters, entropy	40
HRV	NN-intervals/CRG (C3–C4)	Standardized pyHRV suite (Gomes et al., 2019) and custom multi-band/wavelet CRG metrics	132
ECG Stat/Spectral	Continuous single-channel ECG (C3–C4)	Windowed statistical moments and spectral density	130
Cardiocycle	Segmented heartbeat waveforms from ECG	Morphological landmarks (P-Q-R-S-T amplitudes, durations)	272
Total Feature Pool			774

To ensure clarity in the statistical analysis and subsequent classification results, specific notations were adopted for complex derived features. EEG-based metrics are denoted by their source signal: ${\text{EEG}}_{\mathrm{asym}}$ for the asymmetry trace and ${\text{EEG}}_{\mathrm{avg}}$ for the average trace. Technical definitions for the non-standard metrics are as follows.

- Percentage Time Above Threshold (%TAT): The proportion of the epoch during which the signal envelope exceeded a threshold derived from the respective descriptive traces (upper, lower, and median), indicating sustained high-amplitude activity.
- Wavelet Δ Mean Skewness: The skewness of the first derivative (rate of change) of the wavelet approximation levels (e.g., cA4), capturing non-stationary shifts in the signal baseline.
- Spectral Band Classification: Frequency intervals were divided into ranges that correspond to the primary cardiac and artifactual components: T-wave components (1–10,Hz), QRS complex characteristics (10–49,Hz), and high-frequency EMG artifacts ( >50 ,Hz).
- EMG Band Kurtosis ( $5^{th}$ Percentile): The $5^{th}$ percentile of kurtosis values calculated within the high-frequency EMG band ( >50 Hz) across windowed segments; this metric identifies intervals of minimal muscle interference, providing a robust estimate of the background signal quality.
- Autocorrelation Metrics: The mean autocorrelation of the Upper Band ( >10 Hz) was utilized to measure the periodicity and stability of high-frequency components within the ECG.

### 2.4 Model training and evaluation

Features with a linear correlation >0.90 were removed, retaining only the feature in each pair with the highest mutual information. Models—including Gaussian Naive Bayes, Logistic Regression, Support Vector Classifier (SVC), and XGBoost—were hyperparameter-tuned using Bayesian optimization via the BayesSearchCV module from the scikit-optimize library (Scikit-optimize contributors, 2021). This was conducted utilizing a 5-fold patient-wise cross-validation strategy. To ensure the model performed effectively across critical Sarnat scales (e.g., distinguishing between mild and moderate/severe HIE), a custom evaluation metric was employed during training (Equation 9):

$$M=\frac{\sqrt{\max\left(0,F_{1}^{\left(1\right)}-0.5\right)\cdot \max\left(0,F_{1}^{\left(2\right)}-0.5\right)}}{2}$$ (9)

This metric is advantageous as it acts as a “gated” geometric mean; it heavily penalizes any model that fails to exceed a random-chance baseline (0.5) in either target class, ensuring balanced predictive performance. Final testing was conducted using patient-wise leave-one-out cross-validation.

### 2.5 Analytical and visualization frameworks

#### 2.5.1 Morphological barycenters (Soft-DTW)

To visualize the central tendency of heartbeat morphologies across different Sarnat stages, average cardiocycle templates were computed using Soft-DTW barycenters (Cuturi and Blondel, 2017). Unlike Euclidean averaging, which can lead to blurred morphological features when signals are temporally misaligned, the Soft-DTW barycenter provides a robust “mean” in the time-series space by minimizing a smoothed version of the Dynamic Time Warping (DTW) distance. The Soft-DTW distance between two sequences x and y is defined in Equation 10 as:

$$dtw_{\gamma }\left(x,y\right)=-\gamma \log\underset{A\in A}{\sum }e^{-\left\langle A,\Delta \left(x,y\right)\right\rangle /\gamma }$$ (10)

where A is the set of all possible alignment matrices, Δ(x,y) is the cost matrix, and γ>0 is a smoothing parameter. The barycenter z of a set of cardiocycles $\left\{x_{1},\ldots ,x_{n}\right\}$ is then found by solving Equation 11:

$$\underset{z}{\min}\overset{n}{\underset{i=1}{\sum }}w_{i}dtw_{\gamma }\left(z,x_{i}\right)$$ (11)

#### 2.5.2 Feature-level correlation and subgroup analysis

To evaluate macro-scale neuro-cardiac interactions across clinical severity groups, an exhaustive feature-level correlation framework was executed. A comprehensive bivariate correlation matrix was computed across all possible cross-modality pairs, directly evaluating every individual univariate EEG-derived descriptor (including spectral power bands, asymmetric envelopes, and multiscale complexities) against every extracted ECG/HRV-derived marker (including time-domain, frequency-domain, and cardiocycle morphological metrics). Pairwise associations were systematically evaluated using Spearman’s rank correlation coefficient (r) to accommodate potential non-Gaussian feature distributions across the cohort. To identify stage-dependent shifts in autonomic-cortical regulation, the dataset was subsequently stratified by clinical severity (Sarnat Stage 1 vs. Stage 2), and separate sub-group correlation matrices were computed to differentiate physiological dissociation from pathological entrainment trends.

#### 2.5.3 Temporal lag and cross-correlation

To investigate the temporal dynamics of neuro-cardiac interactions, a constrained cross-correlation analysis was performed between the smoothed aEEG upper envelope, denoted as x(t) , and the continuous heart rate (HR) trace, denoted as y(t) , within sliding epoch windows of 300 s. This windowed approach is a well-established framework in network physiology for capturing transient coupling states between distinct organ systems (Bashan et al., 2012; Bartsch et al., 2015). To isolate rapid neuro-cardiac micro-interactions from slower macro-scale metabolic drifts, both signals were linearly detrended and standardized to zero mean and unit variance. The normalized cross-correlation sequence $R_{xy}\left(m\right)$ as a function of discrete lag m was calculated using Equation 12:

$$R_{xy}\left(m\right)=\frac{1}{N}\overset{N-1-m}{\underset{n=0}{\sum }}x\left(n\right)\cdot y\left(n+m\right),\;m\geq 0$$ (12)

where N represents the total number of sample points within the analytical window. To focus strictly on immediate reflexive autonomic responses, the temporal shift (lag) was restricted to a physiologically plausible window of ±20 seconds, consistent with the documented temporal latencies of autonomic neuro-cardiac reflex arcs (Silvani et al., 2016; Equation 13):

$$-\tau_{\max}\leq \frac{m}{f_{s}}\leq \tau_{\max}$$ (13)

where $\tau_{\max}=20$ seconds and $f_{s}=100$ Hz represents the synchronized resampling frequency. The local maximum coupling strength (r) and its corresponding temporal lag $\left(\tau_{\text{best}}\right)$ were extracted within each window by identifying the absolute peak of the valid cross-correlation sequence (Equations 14, 15):

$$\tau_{\text{best}}=\frac{1}{f_{s}}\arg\underset{m}{\max}|R_{xy}\left(m\right)|$$ (14)

$$r=R_{xy}\left(f_{s}\cdot \tau_{\text{best}}\right)$$ (15)

Finally, the temporal lag (seconds) and maximum coupling strength (r) metrics were aggregated at the patient-state level using a non-parametric median operator across their respective long-term recording segments to preserve strict statistical independence during subsequent group-level hypothesis testing.

## 3 Results

### 3.1 Feature significance analysis

To evaluate the predictive capacity of the extracted features, statistical significance and class separation were analyzed using Spearman’s rank correlation coefficient (r) with respect to the clinical Sarnat stage (Stage 1 vs. Stage 2), the Mann-Whitney U test ( p -value), and the Area Under the Receiver Operating Characteristic Curve (AUC). The top features across physiological domains are summarized in Table 4.

**TABLE 4:** Summary of top significant features across physiological domains (Sarnat Stage 1 vs. 2).

Domain	Feature description	Spearman r	MWU p	AUC	Higher for
EEG	${\text{EEG}}_{\mathrm{asym}}$ Upper Envelope %TAT	−0.337	0.001	0.711	Stage 1
	${\text{EEG}}_{\mathrm{asym}}$ Lower Trace Burst Rate	−0.296	0.004	0.685	Stage 1
	${\text{EEG}}_{\mathrm{avg}}$ Peak Spectral Power (0.5–15Hz)	−0.257	0.014	0.661	Stage 1
	${\text{EEG}}_{\mathrm{avg}}$ Raw Burst Rate	0.235	0.024	0.352	Stage 2
HRV	Kurtosis of Interp. CRG Wavelet (cA3)	0.267	0.010	0.332	Stage 2
	FFT LF/HF Ratio	−0.246	0.018	0.654	Stage 1
	VLF Power (Normalized)	0.246	0.018	0.345	Stage 2
ECG (Static)	QRS Band Median Amplitude (10–49 Hz)	0.249	0.017	0.343	Stage 2
	EMG Band Mean Energy ( >50 Hz)	−0.217	0.039	0.636	Stage 1
	T-wave Band Median Amplitude (1–10 Hz)	0.207	0.048	0.369	Stage 2
ECG (Windowed)	Skewness of Wavelet cA4 Δ Mean	0.359	0.0006	0.274	Stage 2
	EMG Band Kurtosis ( $5^{th}$ Percentile)	−0.348	0.0008	0.718	Stage 1
	Upper Band Mean Autocorrelation	0.309	0.003	0.305	Stage 2
Cardiocycles	Q-Peak Absolute Amplitude Kurtosis	0.257	0.014	0.338	Stage 2
	Median T-Wave Duration	−0.222	0.034	0.639	Stage 1
	Median QRS Energy	−0.202	0.053	0.627	Stage 1

Spearman’s r denotes the rank correlation coefficient calculated between each feature and the clinical Sarnat stage.

**FIGURE 4:**
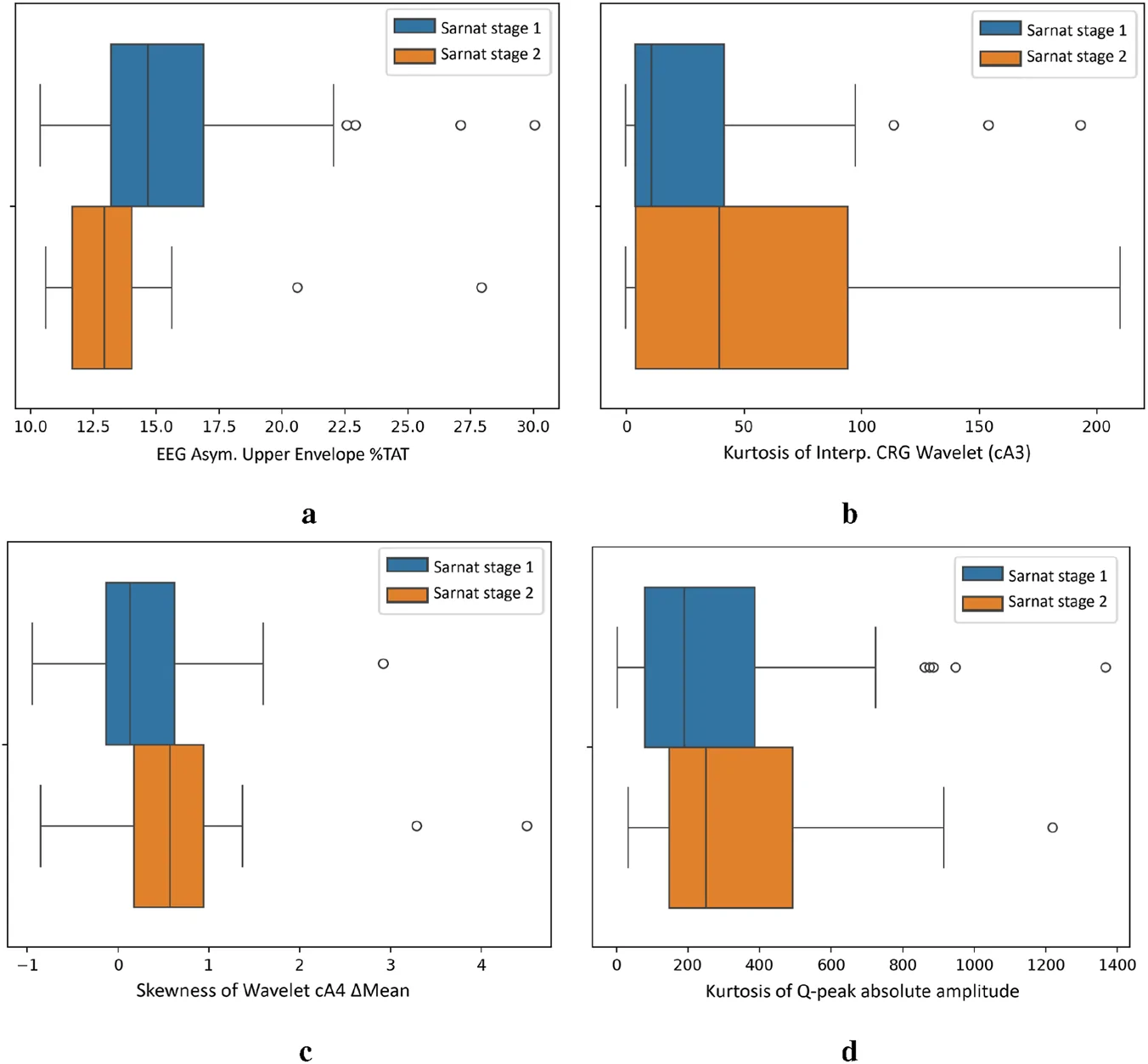
Distribution of representative significant features across patient classes for **(a)** EEG, **(b)** HRV, **(c)** windowed ECG, and **(d)** specific cardiocycle morphology.

The analysis revealed that features distinguishing Sarnat Stage 1 from Stage 2 exhibited domain-specific patterns. For example, EEG high-amplitude trace percentages (e.g., ${\text{EEG}}_{\mathrm{asym}}$ Upper Envelope %TAT) and specific HRV spectral ratios were significantly higher in Stage 1 patients. Conversely, morphological variations in ECG and cardiocycles–specifically increased kurtosis of the wavelet coefficients, QRS segment metrics, and kurtosis of specific peak amplitudes–were generally elevated in Stage 2, suggesting increased signal complexity or irregularity associated with disease progression. Figure 4 highlights the statistical distribution of selected highly significant neurophysiological and cardiovascular features, such as.

1. EEG Asymmetry Upper Envelope %TAT: The percentage of time that the upper envelope of the inter-channel EEG asymmetry signal spends above a clinical threshold of 25 μ V (Time-Above-Threshold).
2. Kurtosis of Interp. CRG Wavelet $\left(cA_{3}\right)$ : The kurtosis calculated from the third-level approximation coefficients $\left(cA_{3}\right)$ derived from the discrete wavelet decomposition of the interpolated cardiorythmogram (CRG), reflecting the heavy-tailed distribution of macro-scale heart rate fluctuations.
3. Skewness of Wavelet $cA_{4}$ Δ Mean: The skewness of the temporal rate of change (first derivative) of the fourth-level wavelet approximation coefficients ( $cA_{4}$ ), quantifying the directional asymmetry of sub-band autonomic variations across the sliding windows.
4. Kurtosis of Q-peak Absolute Amplitude: The kurtosis of the probability distribution of absolute Q-peak amplitudes extracted from the raw ECG waveform, highlighting changes in ventricular depolarization stability between Sarnat stages.

As demonstrated by the distinct group separations in Figure 4, these cross-modal metrics provide clear physiological stratification during the critical transition from mild to moderate encephalopathy.

### 3.2 EEG-based classification

Quantitative EEG (qEEG) and amplitude-integrated EEG (aEEG) features were evaluated on the full cohort ( n=180 ; 118 Stage 1, 62 Stage 2) and on the subset with synchronized ECG ( n=61 ; 47 Stage 1, 14 Stage 2). In the full dataset, the peak grid performance was obtained with the SVC model using a 180 s smoothing window ( $F_{1}=82.21\%$ , accuracy =82.37% ). However, because the SVC demonstrated substantial performance volatility across adjacent window sizes, this specific peak should be treated with caution as a localized hyperparameter anomaly. Conversely, Logistic Regression demonstrated excellent architectural stability across the entire window grid, yielding a reliable average baseline $F_{1}$ of 77.27%. In the ECG-matched subset, Logistic Regression with a 120 s window achieved the highest local result ( $F_{1}=82.69\%$ , accuracy =84.78% ). Relevant features driving these classification boundaries included Hjorth parameters, band powers, entropy measures, burst rate, and aEEG amplitude statistics. A comprehensive breakdown of these model-specific metrics across both cohorts is detailed in Table 5.

**TABLE 5:** EEG model performance (F1) across smoothing windows.

Window length	GaussianNB	XGBoost	LogisticRegression	SVC	Average
Full cohort					
15	60.82%	74.87%	76.24%	74.52%	71.61%
30	64.23%	72.72%	78.31%	76.32%	72.90%
60	61.59%	74.94%	76.97%	69.48%	70.75%
120	62.05%	74.28%	77.96%	74.05%	72.09%
180	67.20%	72.89%	76.43%	82.21%	74.68%
300	60.38%	73.56%	77.70%	71.62%	70.82%
Average	62.71%	73.88%	77.27%	74.70%	72.14%
Subset with ECG					
15	65.84%	63.14%	80.94%	70.77%	70.07%
30	63.52%	72.17%	78.79%	66.89%	71.04%
60	49.93%	80.12%	66.92%	55.48%	65.66%
120	65.15%	68.55%	82.69%	69.29%	70.59%
180	58.92%	66.63%	69.68%	53.78%	65.19%
300	52.02%	75.85%	70.27%	66.09%	69.18%
Average	59.23%	71.08%	74.88%	63.72%	68.62%

**FIGURE 5:**
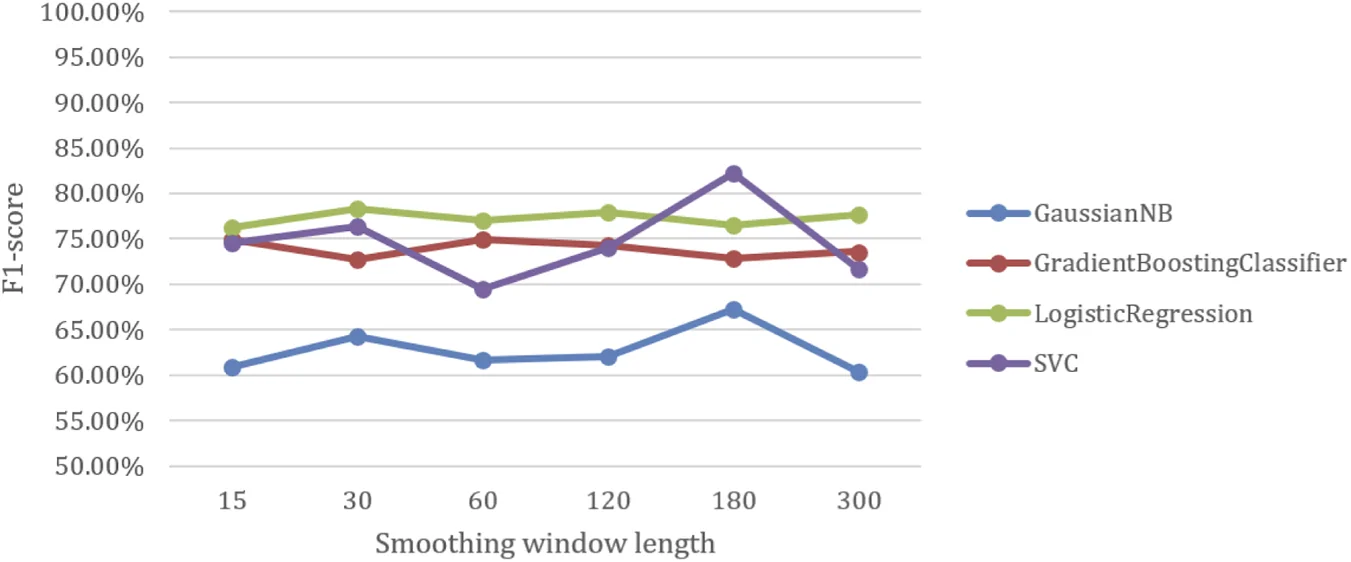
Window length impact on F1-score/accuracy. Total cohort. ECG subgroup.

**FIGURE 6:**
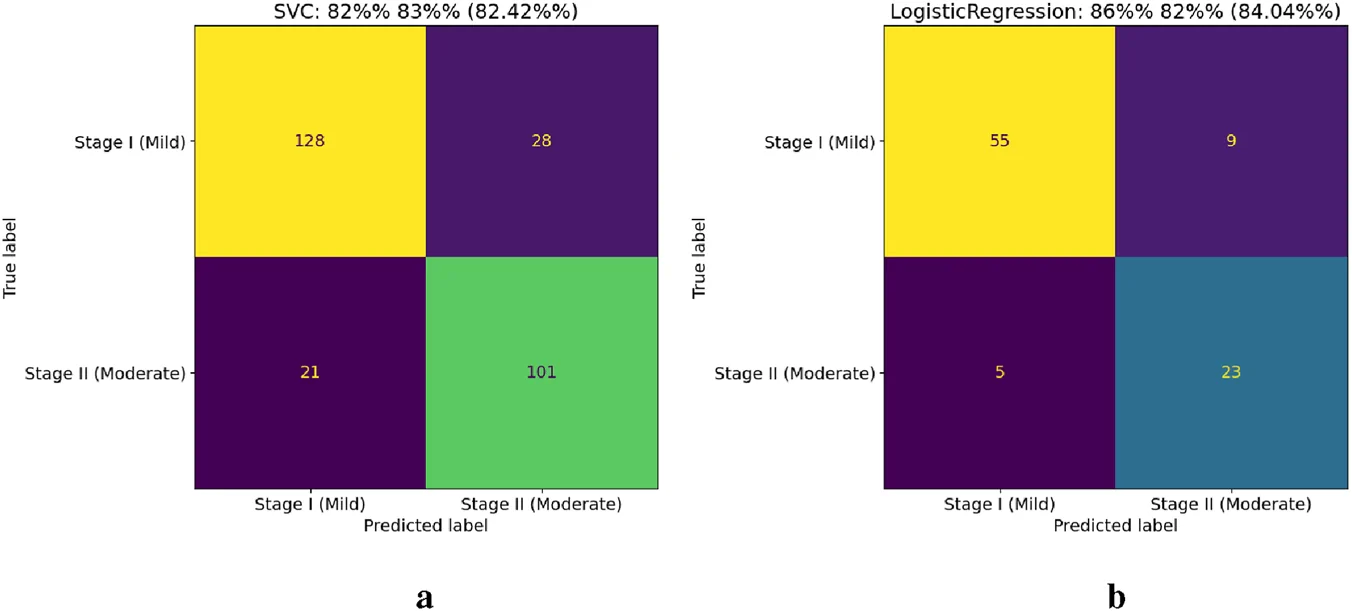
Confusion matrices for the best EEG models on: **(a)** the total cohort of EEG records, and **(b)** subgroup with synchronized ECG.

To evaluate the broader systemic influence of temporal smoothing, performance metrics were aggregated across all models and architectural configurations. As illustrated by the trend analysis in Figure 5, the overall optimal window length was identified as 180 s, producing a mean $F_{1}$ -score of 74.68% and a mean accuracy of 75.00%. The specific class-wise distributions, sensitivities, and localized errors for these leading models are explicitly detailed via the confusion matrices in Figure 6.

### 3.3 HRV classification

Heart rate variability (HRV) features provided moderate discrimination between Sarnat stages, with the highest local grid configuration achieved by the SVC model utilizing the Standardized Feature Set ( $F_{1}=77.96\%$ , Accuracy = 81.52% ). Significant predictors included NN interval statistics, pNN20/pNN50, Sample Entropy (SampEn), the triangular index, and standardized spectral HRV measures.

The performance gap between the extraction methodologies suggests that while the Extended Feature Set provided a more granular description of the signal’s micro-fluctuations, the Standardized Feature Set offered a more robust generalization. This performance difference is likely attributable to the balance between feature dimensionality and signal stability; the standardized suite’s integrated artifact-mitigation protocols minimized the risk of model overfitting, whereas the expansive nature of the extended set introduced greater variance that likely required more stringent feature selection to fully resolve (Table 6).

**TABLE 6:** Classification performance ( $F_{1}$ -scores) across different HRV feature sets and machine learning models.

Feature set	GaussianNB	XGBoost	Log. Reg.	SVC	Average
Extended Feature Set	62.45%	63.14%	60.75%	58.50%	61.21%
Standardized Feature Set	59.03%	57.96%	61.74%	77.96%	64.17%
Average	60.74%	60.55%	61.24%	68.23%	62.69%

### 3.4 ECG statistical, spectral and cardiocycle features

When evaluated on the matched cohort, ECG-derived models demonstrated a diagnostic capacity highly comparable to the EEG-only approaches, showing a minor numerical advantage in peak configurations rather than a definitive statistical divergence. Error analysis reveals that the classification margins between modalities on the minority class are exceptionally narrow, often amounting to a single-neonate difference (e.g., 24 vs. 23 correctly classified Stage 2 records between the top ECG and EEG models). For statistical/spectral ECG features alone, XGBoost with the combined static + windowed set achieved $F_{1}=84.27\%$ and an accuracy of 86.96% .

**FIGURE 7:**
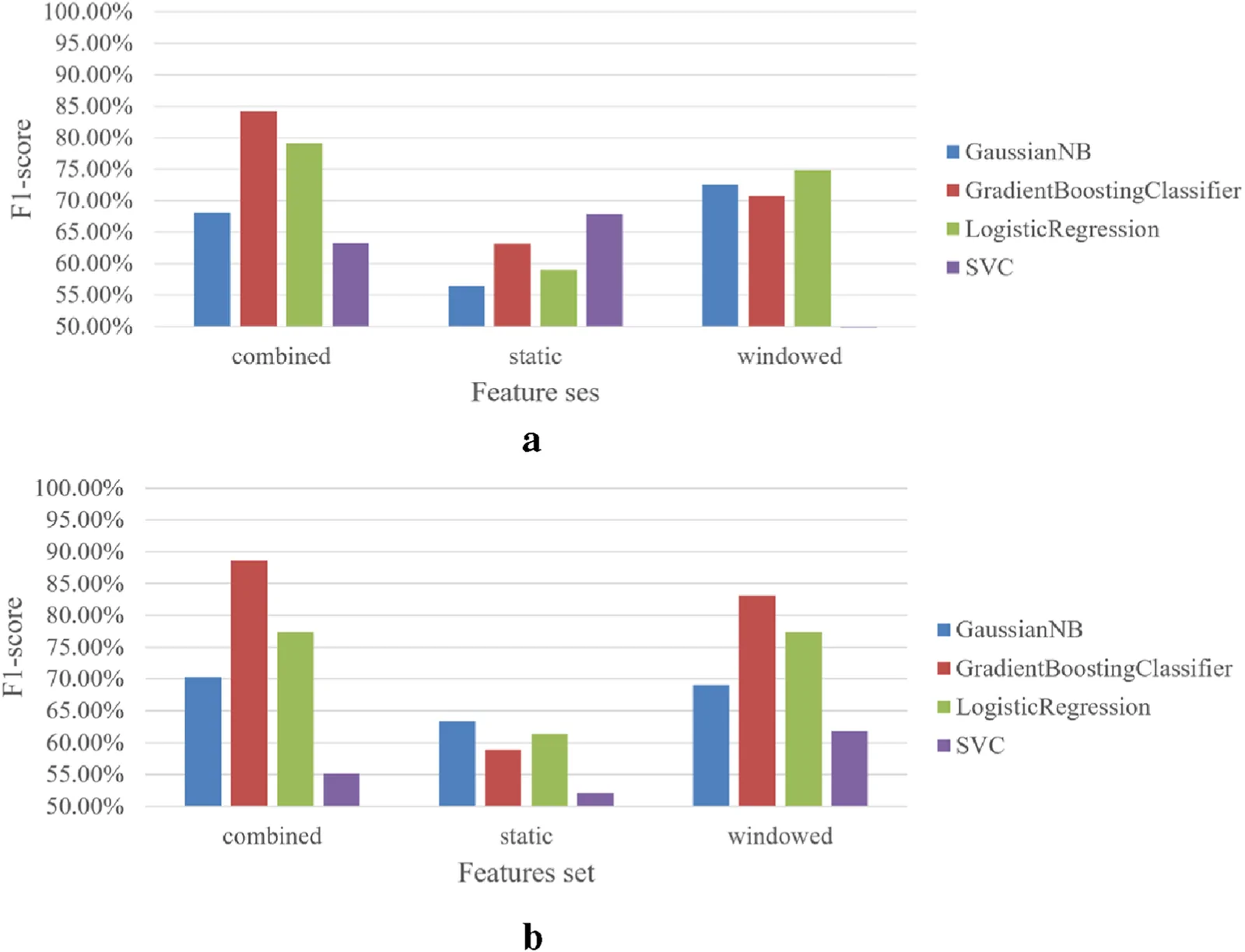
ECG feature-set comparison: **(a)** ECG statistical and spectral features only, **(b)** with cardiocycle features added.

Key features driving these models were T-wave duration/amplitude descriptors and P-wave related metrics, indicating clinically relevant repolarization and conduction dynamics (Table 7; Figure 7).

**TABLE 7:** ECG feature-set comparison (F1-scores).

Features set	GaussianNB	XGBoost	LogisticRegression	SVC	Average
combined	68.13%	84.27%	79.16%	63.23%	73.70%
static	56.36%	63.14%	58.92%	67.85%	61.57%
windowed	72.54%	70.77%	74.82%	48.91%	66.76%
Average	65.68%	72.73%	70.97%	60.00%	67.34%

**FIGURE 8:**
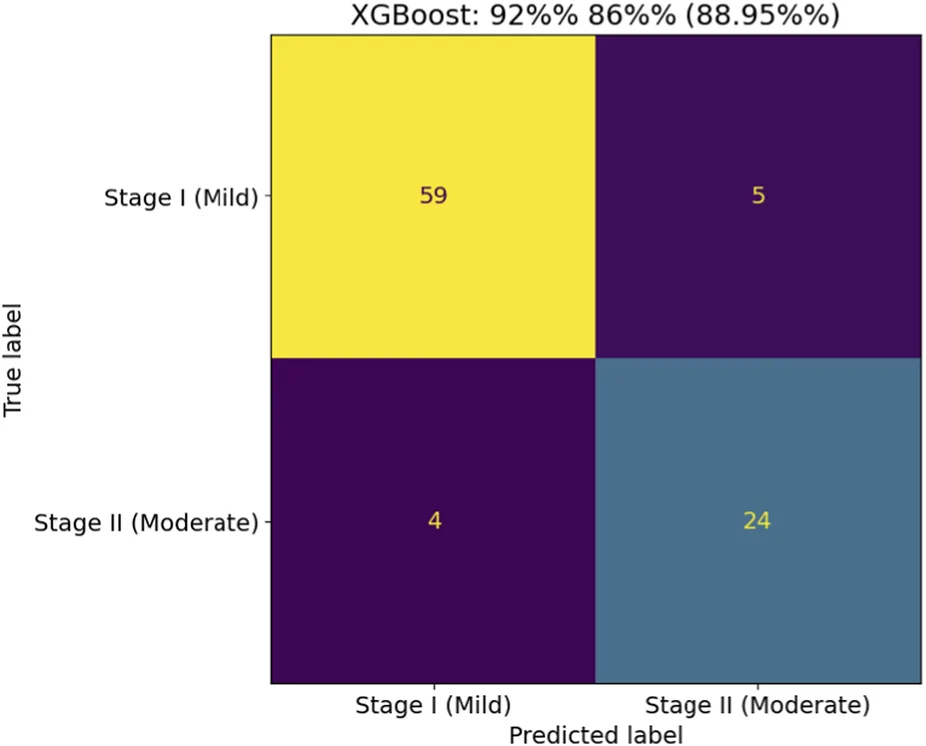
Confusion matrix of best overall ECG model.

Utilizing these highly discriminative features, the highest nominal performance of the study (Figure 8) was obtained by combining cardiocycle morphology with ECG statistical/spectral features using XGBoost ( $F_{1}=88.56\%$ , accuracy =90.22% ). While this configuration represents an optimized upper bound for multi-modal feature fusion, the model maintained a stable baseline average accuracy of 72.73% across the broader parameter grid. Given the small sample size of the matched cohort and the lack of nested validation loops, these peak metrics are treated as exploratory benchmarks to be formally evaluated on larger, multi-center datasets.

### 3.5 Morphological visualization

**FIGURE 9:**
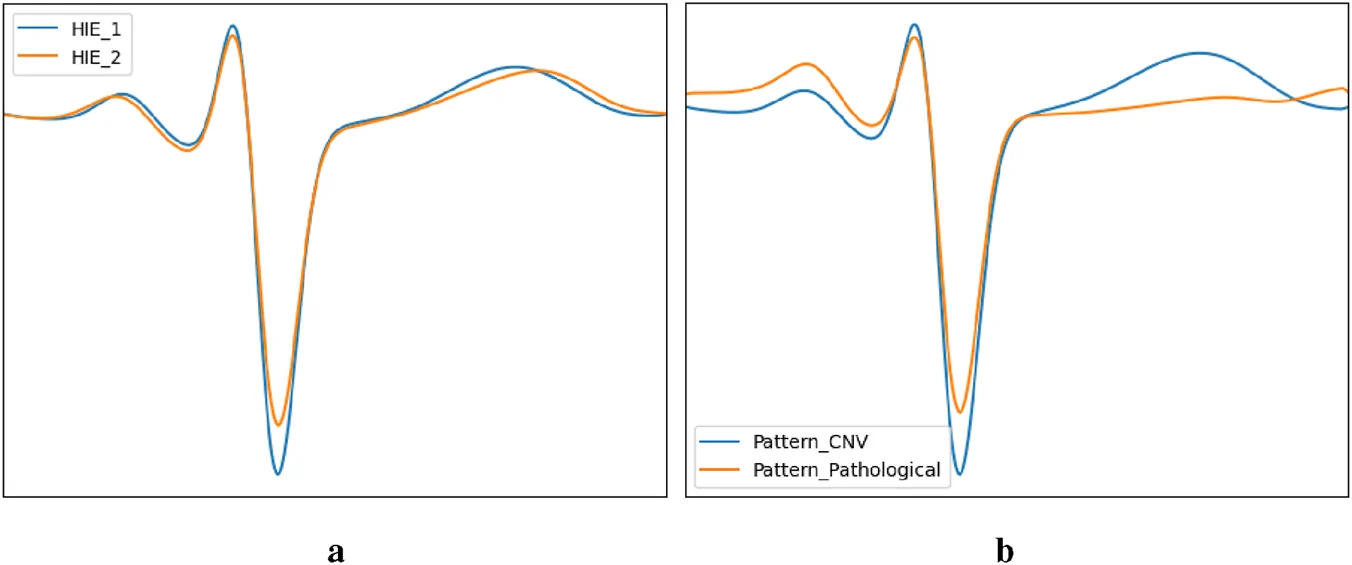
Typical cardiocycles comparing: **(a)** Sarnat stages 1 and 2, **(b)** CNV and pathological patterns.

Following the Soft-DTW extraction protocol (Section 2.5.1), barycenters of typical cardiocycles showed limited visual separation between Sarnat stages 1 and 2, but clearer differences between normal and pathological groups, particularly reduced or inverted T-wave morphology in abnormal states (Figure 9).

Soft-DTW barycenters of typical cardiocycles showed limited visual separation between Sarnat stages 1 and 2, but clearer differences between normal and pathological groups, particularly reduced or inverted T-wave morphology in abnormal states (Figure 9).

### 3.6 Heart-brain correlation analysis and Sarnat Stage subgrouping

Significant baseline correlations were observed between cerebral activity and cardiovascular metrics across the entire cohort. However, subgroup analysis revealed stark, diverging trajectories in coupling strength as encephalopathy progressed from Stage 1 (mild) to Stage 2 (moderate) (Table 8). Two distinct coupling behaviors emerged. First, a loss of healthy synchronization (neuro-cardiac dissociation) was observed in complexity and envelope metrics. For example, $EEG_{\mathrm{avg}}$ median envelope and median QRS kurtosis were strongly coupled in Stage 1 ( r=0.53 , p<0.001 ) but lost all significant correlation in Stage 2 ( r=0.16 , p=0.42 ). Conversely, a pattern of pathological entrainment emerged between structural brain rhythms and specific morphological abnormalities. $EEG_{\mathrm{avg}}$ δ -power and ECG Q-peak amplitude skewness showed no significant relationship in Stage 1 ( r=−0.11 , p=0.38 ), but became highly coupled in Stage 2 ( r=−0.69 , p<0.001 ). These findings suggest that the transition from mild to moderate HIE is characterized not simply by a global loss of connectivity, but by a shift from adaptable autonomic regulation to rigid, stress-driven synchrony.

**TABLE 8:** Diverging trajectories of EEG–ECG/HRV coupling strength across Sarnat Stages.

Coupling behavior	Paired features (EEG and ECG/HRV)	Overall r	Stage 1 r	Stage 2 r
Neuro-Cardiac Dissociation (Coupling breaks down in moderate HIE)				
ECG (Windowed)	${\text{EEG}}_{\mathrm{asym}}$ Upper Envelope and Median QRS Kurtosis	$0.51^{***}$	$0.58^{***}$	$0.26^{ns}$
ECG (Windowed)	${\text{EEG}}_{\mathrm{avg}}$ Median Envelope and Median QRS Kurtosis	$0.44^{***}$	$0.53^{***}$	$0.16^{ns}$
HRV	${\text{EEG}}_{\mathrm{avg}}$ Median Envelope and HRV Wavelet Std. Dev. (cA3)	$-0.43^{***}$	$-0.53^{***}$	$-0.19^{ns}$
ECG (Windowed)	${\text{EEG}}_{\mathrm{asym}}$ Complexity and Median QRS Kurtosis	$-0.46^{***}$	$-0.56^{***}$	$-0.20^{ns}$
Pathological Entrainment (Coupling emerges in moderate HIE)				
HRV	${\text{EEG}}_{\mathrm{asym}}$ Upper Envelope and HRV LF Power	$0.52^{***}$	$0.27^{*}$	$0.67^{***}$
HRV	${\text{EEG}}_{\mathrm{asym}}$ Mobility and HRV LF Power	$0.46^{***}$	$0.22^{ns}$	$0.62^{***}$
Cardiocycles	${\text{EEG}}_{\mathrm{avg}}$ θ -Power (2–4 Hz) and Q-Peak Amplitude Skewness	$-0.42^{***}$	$-0.19^{ns}$	$-0.66^{***}$
Cardiocycles	${\text{EEG}}_{\mathrm{avg}}$ δ -Power (0.5–2 Hz) and Q-Peak Amplitude Skewness	$-0.40^{***}$	$-0.11^{ns}$	$-0.69^{***}$
HRV	${\text{EEG}}_{\mathrm{asym}}$ Upper Envelope and HRV Wavelet Std. Dev. (cA3)	$-0.49^{***}$	$-0.21^{ns}$	$-0.70^{***}$

Significance levels:^*n s*^ p≥0.05 ,^*^ p<0.05 ,^**^ p<0.01 ,^***^ p<0.001 .

#### 3.6.1 Temporal lag and coupling strength analysis

Based on the constrained cross-correlation protocol (Section 2.5.3), Table 9 summarizes the descriptive statistics for temporal lag and coupling strength stratified by Sarnat stage and baseline aEEG pattern. Analysis of sex-based differences yielded negligible variations in the broader cohort and was excluded from the primary evaluation to maintain focus on clinical severity markers.

**TABLE 9:** Descriptive statistics for temporal lag and coupling strength (patient-level aggregation).

Temporal lag (seconds)							
Group	n	Mean	Median	SD	IQR	10th Pct	90th Pct
*Sarnat Stage*							
Stage 1	47	−1.35	−2.08	4.20	4.25	−5.68	3.95
Stage 2	14	−1.62	−1.35	2.57	2.51	−4.38	1.05
*EEG Pattern*							
Normal (CNV)	73	−1.04	−1.50	4.61	4.10	−6.16	4.90
Abnormal	10	−1.89	−2.30	3.89	2.03	−4.86	2.89

Maximum coupling strength (r)							
Group	n	Mean	Median	SD	IQR	10th Pct	90th Pct
*Sarnat Stage*							
Stage 1	47	0.19	0.19	0.21	0.24	−0.00	0.46
Stage 2	14	0.23	0.21	0.16	0.18	0.03	0.47
*EEG Pattern*							
Normal (CNV)	73	0.21	0.22	0.19	0.24	0.01	0.46
Abnormal	10	0.05	0.09	0.17	0.19	−0.14	0.23

**FIGURE 10:**
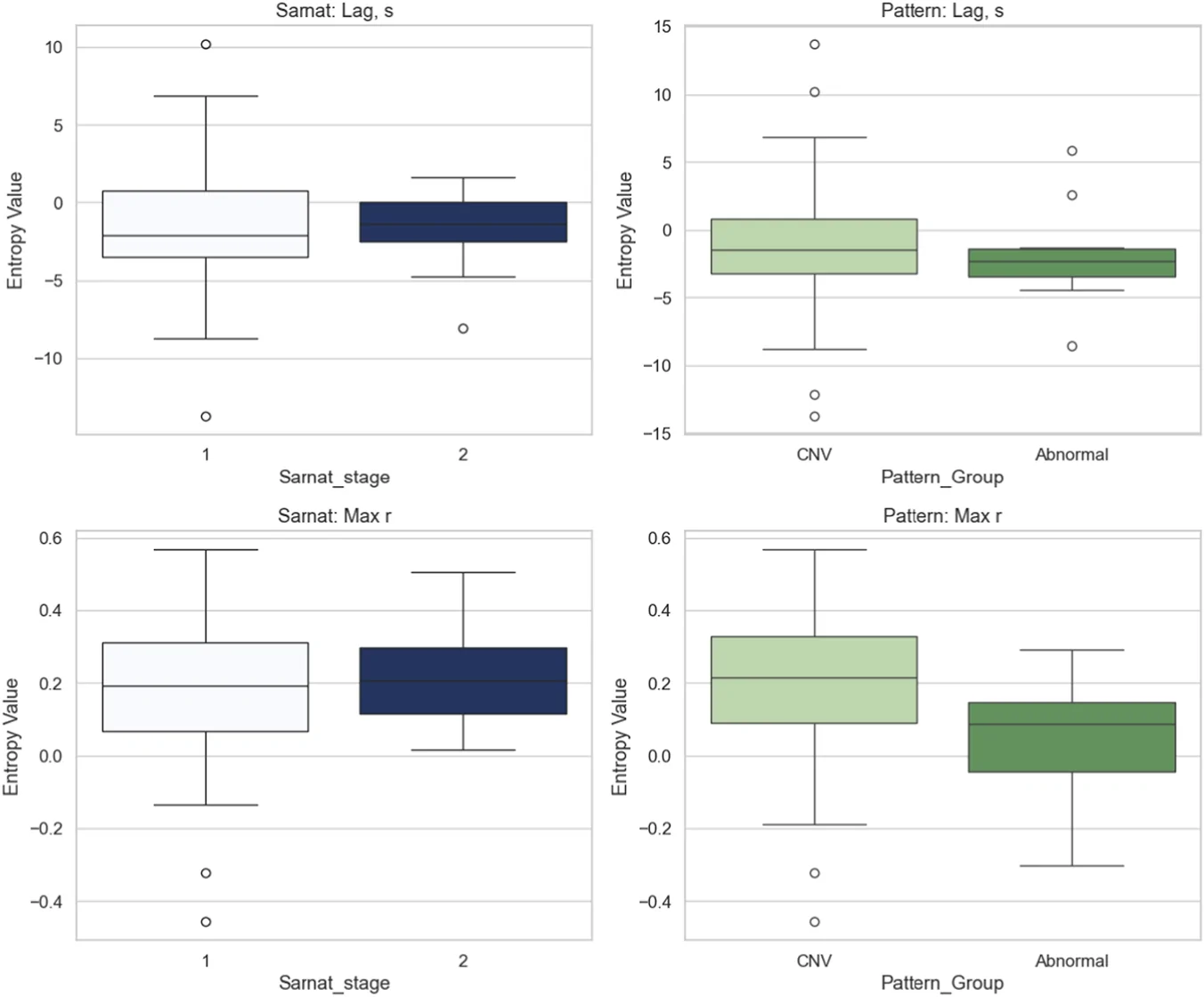
Boxplots describing the distribution of temporal lag (top row) and maximum coupling strength (bottom row) for the patient-aggregated cohort.

Hypothesis testing revealed a statistically significant difference in maximum coupling strength (r) between neonates with healthy and suppressed aEEG patterns. While other metrics did not reach standard significance thresholds (p≥0.05) , two physiological trends emerged (Figure 10). First, temporal lag demonstrated a shift in magnitude based on HIE severity. Stage 1 neonates showed a median negative lag of −2.08 s, indicating that cardiovascular rate changes typically preceded cortical envelope fluctuations. In Stage 2 neonates, this lead-time was reduced to a median of −1.35 s, suggesting a potentially less responsive or more synchronized state associated with moderate encephalopathy.

Second, the maximum coupling strength (r) exhibited a significant decline associated with baseline aEEG background suppression. Neonates with a healthy CNV pattern maintained a median coupling strength of r=0.22 , whereas those with abnormal patterns (e.g., burst suppression or low voltage) dropped to a median of r=0.09 . This represents an approximately 60% reduction in neuro-cardiac synchronization strength in the abnormal group ( p=0.015 , AUC=0.74 ), strongly supporting the hypothesis that electrocortical integrity is essential for robust physiological coupling in the neonatal period (Table 10).

**TABLE 10:** Mann-Whitney U test results for patient-grouped temporal and coupling metrics.

Comparison	Metric	$n_{1},n_{2}$	U	AUC	p -value	Significance
Sarnat Stage (1 vs. 2)	Lag (s)	47, 14	314.0	0.48	0.804	False
	Max r	47, 14	307.0	0.47	0.712	False
Pattern (CNV vs. Abn.)	Lag (s)	73, 10	421.0	0.58	0.438	False
	Max r	73, 10	540.0	0.74	0.015	True

## 4 Discussion

The results indicate that cardiovascular signals contain robust information for automated HIE grading. The comparable performance of ECG-based models relative to EEG-based approaches suggests that the heart integrates systemic hypoxic, metabolic, and autonomic stress, reflecting the global severity of the condition. Crucially, automated ECG assessment is not intended to replace the evaluation of HIE without EEG; rather, it provides a vital complementary component for staging. Because HIE is a complex, systemic pathological condition affecting the entire organism rather than the central nervous system in isolation, ECG metrics uniquely capture the impairment of cardiac function and its functional coupling with the brain (Rezaei et al., 2026). The repeated association between EEG slow-wave activity and HRV/ECG metrics is physiologically plausible. Neonatal slow oscillations are linked to cortical integrity, while autonomic output is modulated through the central autonomic network. Hypoxic-ischemic injury may therefore disrupt both brain rhythms and cardiac variability simultaneously, underscoring the importance of assessing this brain-heart axis (Bersani et al., 2021; Shah et al., 2025).

Beyond global correlations, the windowed analysis revealed a dynamic shift in heart-brain interaction as encephalopathy worsened. A key finding of this study is the significant reduction in neuro-cardiac synchronization associated with baseline aEEG suppression. Neonates with healthy CNV patterns maintained a median coupling strength of r=0.22 , whereas the abnormal group exhibited a 60% decline to r=0.09 (p=0.015) . This suggests that electrocortical integrity is a fundamental prerequisite for robust physiological coupling, and its breakdown serves as a quantifiable marker of neurological compromise.

The temporal lag analysis further characterizes this decoupling. In Stage 1 neonates, we observed a median negative lag of −2.08 s, indicating a “heart-leads-brain” sequence where cardiovascular fluctuations precede changes in the electrocortical envelope. This likely reflects a healthy, responsive autonomic reflex arc. However, in Stage 2 neonates, this lead-time was reduced to −1.35 s. While this shift did not reach statistical significance (p=0.804) due to high inter-patient variability, it points toward a reorganization of the autonomic reflex, potentially moving toward a less adaptable, more rigid synchronization state as HIE severity increases.

In Stage 1, metrics of EEG complexity were linked to autonomic modulation, reflecting a healthy Central Autonomic Network (CAN). The dissociation observed in the Abnormal pattern group aligns with clinical observations of moderate HIE, in which increasing cortical depression and brainstem edema cause the cardiovascular system to “uncouple” from top-down neurological control. Identifying this transition from adaptable regulation to pathological dissociation provides a powerful, multi-modal mechanistic marker that neither EEG nor ECG could offer in isolation. From the conceptual perspective of Network Physiology, this shift represents a critical network phase transition (Bashan et al., 2012; Bartsch et al., 2015). While healthy physiological states are sustained by flexible, non-linear interactions across multiple organ systems to dynamically adapt to stress, acute hypoxic-ischemic injury degrades this global network topology. The loss of top-down cortical control ultimately forces the brain-heart network to collapse from an adaptable, high-entropy state into a highly rigid, pathological synchronization.

Furthermore, the pilot nature of this cohort introduces structural imbalances; specifically, the Sarnat Stage 2 sub-cohort exhibited a heavy sex imbalance (12 males vs. Two females). While post-hoc exploratory analysis did not reveal statistically significant sex-driven differences in the extracted coupling metrics, a larger, prospectively matched cohort is required to completely rule out sex as a biological variable. Additionally, while our pipeline proved resilient to real-world NICU noise through robust windowed averaging, formal signal quality indices (SQI) should be integrated into future real-time monitoring iterations.

While this study provides robust evidence of neuro-cardiac impairment in HIE, we acknowledge a terminological limitation. Our approach utilizes standard univariate feature extraction from independent cortical and cardiac domains, subsequently evaluating their feature-level associations. While this highlights significant systemic breakdown, it does not measure instantaneous, continuous dynamical coupling (such as transfer entropy or phase synchronization), which remains an important avenue for future real-time monitoring research.

Moving forward, future research will focus on employing nonlinear coupling measures, such as mutual information and transfer entropy, to more precisely characterize the complex, multi-scale interactions of the neuro-cardiac axis across larger, multi-center validation cohorts.

## 5 Conclusion

In this study, we investigated the quantitative relationship between electrocortical activity and cardiovascular dynamics in neonates with hypoxic-ischemic encephalopathy. By employing windowed cross-correlation and patient-level aggregation, we identified that HIE severity is characterized by a significant breakdown in neuro-cardiac coupling strength rather than a simple global loss of connectivity, reflecting a pathological reorganization of the neonatal brain-heart network topology. Our findings highlight that neonates with abnormal aEEG patterns undergo a statistically significant 60% reduction in coupling strength compared to those with healthy background activity (p=0.015) .

These interaction-based metrics provide a robust multi-modal framework for HIE assessment, offering superior physiological insight compared to isolated unimodal analysis. Because ECG monitoring is ubiquitous in neonatal intensive care, the integration of these coupling markers into existing monitoring systems could enhance diagnostic precision, especially in settings where continuous EEG interpretation is not immediately available. Future work involving larger, multi-center cohorts is required to investigate the clinical validity of exploratory temporal lead-time dynamics and to refine these integrated coupling features for real-time clinical decision support.

## Data Availability

The original contributions presented in the study are included in the article/Supplementary Material, further inquiries can be directed to the corresponding author.
