# Supplementary material for: Quantitative assessment of heart-brain coupling in neonates with hypoxic-ischemic encephalopathy
Source: Front Netw Physiol. 2026 Sep 7;6:1889331. doi: 10.3389/fnetp.2026.1889331 (PMC13590696; doi:10.3389/fnetp.2026.1889331)
Supplement: Supplementary file 1 [file Table1.docx]

**Table 1.** Key Studies of Neonatal EEG–ECG Feature Correlation.

| **Study (Year)** | **Population & Signals** | **N** | **Correlated Features** | **Main Result (Effect Size)** | **DOI** |
| --- | --- | --- | --- | --- | --- |
| Greene et al. (2007) | 10 term neonates; EEG (7-12ch) + ECG (NICU data) | 10 | Seizure epochs (EEG) vs HR changes | Combined EEG+ECG detector GDR ∼98% (vs 81% EEG-only) (8) | [10.1016/…](https://doi.org/10.1016/j.clinph.2007.03.022) |
| Doyle et al. (2008) | 12 neonates; EEG + ECG (seizure monitoring) | 12 | EEG RMS vs iHR change | 100% patients had EEG--HR correlation during seizures (3) | [10.1109/…](https://doi.org/10.1109/IEMBS.2008.4650333) |
| Pfurtscheller et al. (2005) | Preterm infants (mean CA=36w); EEG (tracé alternant) + HR | ∼9 | EEG slow-wave bursts vs HR accel. | Slow-wave bursts tightly coincide with transient HR accelerations (2) | [10.1016/…](https://doi.org/10.1016/j.braindev.2005.01.007) |
| Goulding et al. (2015) | Term HIE infants; EEG grade + ECG | 46 | HRV (SDNN, LF, HF, etc.) vs EEG grade | HRV metrics negatively correlated with EEG severity (*r* ≈ -0.48 to -0.67) (4) | [10.1038/…](https://doi.org/10.1038/pr.2015.28) |
| Pavel et al. (2023) | Term HIE infants; EEG grade + ECG | 120 | HRV (time, freq, entropy) | HRV model AUC=0.84 for predicting EEG grade (5) | [10.3389/…](https://doi.org/10.3389/fped.2022.1016211) |
| Frassineti et al. (2021) | Neonatal seizures; ECG (Helsinki NICU dataset) | 52 | HRV multiscale entropy | Seizure vs non-seizure groups differ in HRV entropy (*p*<0.05) (7) | [10.3390/…](https://doi.org/10.3390/bioengineering8090122) |
| Govindan et al. (2026) | Preterm sleep study; EEG + ECG | 68 | Sleep state (EEG δ-power vs HR range) | QS: ↑ δ-power vs AS, distinct HR range (AUC ≈ 0.88) (6) | [10.1038/…](https://doi.org/10.1038/s41598-025-31873-7) |

**Abbreviations**: SDNN: standard deviation of NN intervals; LF/HF: low-frequency/high-frequency; iHR: instantaneous heart rate; CA: conceptional age; GDR: gross detection rate; RMS: root-mean-square.

**Table 2.** Dataset distribution across Sarnat stages, highlighting the subset of records containing synchronous ECG data.

| **Sarnat Stage** | **Total EEG Records (Patients)** | **Subgroup with ECG (Patients)** |
| --- | --- | --- |
| Stage 0 | 15 (14) | 11 (11) |
| Stage 1 | 156 (118) | 64 (47) |
| Stage 2 | 122 (62) | 28 (14) |
| Stage 3 | 27 (7) | 5 (1) |

**Table 3.** Summary of the extracted feature space across physiological domains.

| **Feature Domain** | **Source Signal / Channels** | **Primary Extraction Framework / References** | **Total Features** |
| --- | --- | --- | --- |
| **EEG (aEEG)** | Derived traces (EEG*_avg_*, EEG*_asym_*) | Statistical descriptors of 4 envelope streams | 200 |
| **EEG (qEEG)** | Derived traces (EEG*_avg_*, EEG*_asym_*) | Spectral power bands, Hjorth parameters, entropy | 40 |
| **HRV** | NN-intervals / CRG (C3-C4) | Standardized pyHRV suite ( 14) and custom multi-band/wavelet CRG metrics | 132 |
| **ECG Stat/Spectral** | Continuous single-channel ECG (C3-C4) | Windowed statistical moments and spectral density | 130 |
| **Cardiocycle** | Segmented heartbeat waveforms from ECG | Morphological landmarks (P-Q-R-S-T amplitudes, durations) | 272 |
| **Total Feature Pool** |  |  | **774** |

**Table 4.** Summary of top significant features across physiological domains (Sarnat Stage 1 vs. 2). Spearman's *r* denotes the rank correlation coefficient calculated between each feature and the clinical Sarnat stage.

| **Domain** | **Feature Description** | **Spearman** *r* | **MWU** *p* | **AUC** | **Higher for** |
| --- | --- | --- | --- | --- | --- |
| **EEG** | EEG*_asym_* Upper Envelope %TAT | -0.337 | 0.001 | 0.711 | Stage 1 |
|  | EEG*_asym_* Lower Trace Burst Rate | -0.296 | 0.004 | 0.685 | Stage 1 |
|  | EEG*_avg_* Peak Spectral Power (0.5-15 Hz) | -0.257 | 0.014 | 0.661 | Stage 1 |
|  | EEG*_avg_* Raw Burst Rate | 0.235 | 0.024 | 0.352 | Stage 2 |
| **HRV** | Kurtosis of Interp. CRG Wavelet (cA3) | 0.267 | 0.01 | 0.332 | Stage 2 |
|  | FFT LF/HF Ratio | -0.246 | 0.018 | 0.654 | Stage 1 |
|  | VLF Power (Normalized) | 0.246 | 0.018 | 0.345 | Stage 2 |
| **ECG (static)** | QRS Band Median Amplitude (10-49 Hz) | 0.249 | 0.017 | 0.343 | Stage 2 |
|  | EMG Band Mean Energy (>50 Hz) | -0.217 | 0.039 | 0.636 | Stage 1 |
|  | T-wave Band Median Amplitude (1-10 Hz) | 0.207 | 0.048 | 0.369 | Stage 2 |
| **ECG (windowed)** | Skewness of Wavelet cA4 ∆Mean | 0.359 | 0.0006 | 0.274 | Stage 2 |
|  | EMG Band Kurtosis (5^th^ Percentile) | -0.348 | 0.0008 | 0.718 | Stage 1 |
|  | Upper Band Mean Autocorrelation | 0.309 | 0.003 | 0.305 | Stage 2 |
| **Cardiocycles** | Q-Peak Absolute Amplitude Kurtosis | 0.257 | 0.014 | 0.338 | Stage 2 |
|  | Median T-Wave Duration | -0.222 | 0.034 | 0.639 | Stage 1 |
|  | Median QRS Energy | -0.202 | 0.053 | 0.627 | Stage 1 |

**Table 5.** EEG model performance (F1) across smoothing windows.

| **Full cohort** | | | | | |  |
| --- | --- | --- | --- | --- | --- | --- |
| **Window length** | **GaussianNB** | **XGBoost** | **LogisticRegression** | **SVC** | **Average** |  |
| 15 | 60.82% | 74.87% | 76.24% | 74.52% | 71.61% |  |
| 30 | 64.23% | 72.72% | 78.31% | 76.32% | 72.90% |  |
| 60 | 61.59% | 74.94% | 76.97% | 69.48% | 70.75% |  |
| 120 | 62.05% | 74.28% | 77.96% | 74.05% | 72.09% |  |
| 180 | 67.20% | 72.89% | 76.43% | 82.21% | 74.68% |  |
| 300 | 60.38% | 73.56% | 77.70% | 71.62% | 70.82% |  |
| **Average** | 62.71% | 73.88% | 77.27% | 74.70% | 72.14% |  |
|  | | | | | |  |
| **Subset with ECG** | | | | | |  |
| **Window length** | **GaussianNB** | **XGBoost** | **LogisticRegression** | **SVC** | **Average** |  |
| 15 | 65.84% | 63.14% | 80.94% | 70.77% | 70.07% |  |
| 30 | 63.52% | 72.17% | 78.79% | 66.89% | 71.04% |  |
| 60 | 49.93% | 80.12% | 66.92% | 55.48% | 65.66% |  |
| 120 | 65.15% | 68.55% | 82.69% | 69.29% | 70.59% |  |
| 180 | 58.92% | 66.63% | 69.68% | 53.78% | 65.19% |  |
| 300 | 52.02% | 75.85% | 70.27% | 66.09% | 69.18% |  |
| **Average** | 59.23% | 71.08% | 74.88% | 63.72% | 68.62% |  |

**Table 6.** Classification performance (F1-scores) across different HRV feature sets and machine learning models.

| **Feature Set** | **GaussianNB** | **XGBoost** | **Log. Reg.** | **SVC** | **Average** |
| --- | --- | --- | --- | --- | --- |
| Extended Feature Set | 62.45% | 63.14% | 60.75% | 58.50% | 61.21% |
| Standardized Feature Set | 59.03% | 57.96% | 61.74% | 77.96% | 64.17% |
| **Average** | 60.74% | 60.55% | 61.24% | 68.23% | 62.69% |

**Table 7.** ECG feature-set comparison (F1-scores).

| **Features set** | **GaussianNB** | **XGBoost** | **LogisticRegression** | **SVC** | **Average** |
| --- | --- | --- | --- | --- | --- |
| combined | 68.13% | 84.27% | 79.16% | 63.23% | 73.70% |
| static | 56.36% | 63.14% | 58.92% | 67.85% | 61.57% |
| windowed | 72.54% | 70.77% | 74.82% | 48.91% | 66.76% |
| **Average** | 65.68% | 72.73% | 70.97% | 60.00% | 67.34% |

**Table 8.** Diverging trajectories of EEG--ECG/HRV coupling strength across Sarnat Stages.

| **Coupling Behavior** | **Paired Features (EEG & ECG/HRV)** | **Overall** *r* | **Stage 1** *r* | **Stage 2** *r* |
| --- | --- | --- | --- | --- |
| **Neuro-Cardiac Dissociation** (Coupling breaks down in moderate HIE) | | | | |
| ECG (Windowed) | EEG_asym_ Upper Envelope & Median QRS Kurtosis | 0.51^***^ | 0.58^***^ | 0.26^ns^ |
| ECG (Windowed) | EEG_avg_ Median Envelope & Median QRS Kurtosis | 0.44^***^ | 0.53^***^ | 0.16^ns^ |
| HRV | EEG_avg_ Median Envelope & HRV Wavelet Std. Dev. (cA3) | -0.43^***^ | -0.53^***^ | -0.19^ns^ |
| ECG (Windowed) | EEG_asym_ Complexity & Median QRS Kurtosis | -0.46^***^ | -0.56^***^ | -0.20^ns^ |
| **Pathological Entrainment** (Coupling emerges in moderate HIE) | | | | |
| HRV | EEG_asym_ Upper Envelope & HRV LF Power | 0.52^***^ | 0.27^*^ | 0.67^***^ |
| HRV | EEG_asym_ Mobility & HRV LF Power | 0.46^***^ | 0.22^ns^ | 0.62^***^ |
| Cardiocycles | EEG_avg_ θ-Power (2-4 Hz) & Q-Peak Amplitude Skewness | -0.42^***^ | -0.19^ns^ | -0.66^***^ |
| Cardiocycles | EEG_avg_ δ-Power (0.5-2 Hz) & Q-Peak Amplitude Skewness | -0.40^***^ | -0.11^ns^ | -0.69^***^ |
| HRV | EEG_asym_ Upper Envelope & HRV Wavelet Std. Dev. (cA3) | -0.49^***^ | -0.21^ns^ | -0.70^***^ |
| **Significance levels:** ^ns^*p* ≥ 0.05, ^*^*p* < 0.05, ^**^*p* < 0.01, ^***^*p* < 0.001. | | | | |

**Table 9.** Descriptive statistics for temporal lag and coupling strength (patient-level aggregation).

| **Temporal Lag (seconds)** | | | | | | | |
| --- | --- | --- | --- | --- | --- | --- | --- |
| **Group** | *n* | **Mean** | **Median** | **SD** | **IQR** | **10th Pct** | **90th Pct** |
| *Sarnat Stage* | | | | | | | |
| Stage 1 | 47 | -1.35 | -2.08 | 4.2 | 4.25 | -5.68 | 3.95 |
| Stage 2 | 14 | -1.62 | -1.35 | 2.57 | 2.51 | -4.38 | 1.05 |
| *EEG Pattern* | | | | | | | |
| Normal (CNV) | 73 | -1.04 | -1.5 | 4.61 | 4.1 | -6.16 | 4.9 |
| Abnormal | 10 | -1.89 | -2.3 | 3.89 | 2.03 | -4.86 | 2.89 |
| **Maximum Coupling Strength (***r***)** | | | | | | | |
| **Group** | *n* | **Mean** | **Median** | **SD** | **IQR** | **10th Pct** | **90th Pct** |
| *Sarnat Stage* | | | | | | | |
| Stage 1 | 47 | 0.19 | 0.19 | 0.21 | 0.24 | 0 | 0.46 |
| Stage 2 | 14 | 0.23 | 0.21 | 0.16 | 0.18 | 0.03 | 0.47 |
| *EEG Pattern* | | | | | | | |
| Normal (CNV) | 73 | 0.21 | 0.22 | 0.19 | 0.24 | 0.01 | 0.46 |
| Abnormal | 10 | 0.05 | 0.09 | 0.17 | 0.19 | -0.14 | 0.23 |

**Table 10.** Mann-Whitney U test results for patient-grouped temporal and coupling metrics.

| **Comparison** | **Metric** | *n_1_, n_2_* | **U** | **AUC** | *p***-value** | **Significance** |
| --- | --- | --- | --- | --- | --- | --- |
| Sarnat Stage (1 vs 2) | Lag (s) | 47, 14 | 314 | 0.48 | 0.804 | False |
|  | Max r | 47, 14 | 307 | 0.47 | 0.712 | False |
| Pattern (CNV vs Abn.) | Lag (s) | 73, 10 | 421 | 0.58 | 0.438 | False |
|  | Max r | 73, 10 | 540 | 0.74 | 0.015 | True |
